# Age-Dependent Vulnerability to Oxidative Stress of Postnatal Rat Pyramidal Motor Cortex Neurons

**DOI:** 10.3390/antiox9121307

**Published:** 2020-12-19

**Authors:** Livia Carrascal, Ella Gorton, Ricardo Pardillo-Díaz, Patricia Perez-García, Ricardo Gómez-Oliva, Carmen Castro, Pedro Nunez-Abades

**Affiliations:** 1 Departament of Physiology, Pharmacy School, University of Seville, 41012 Seville, Spain; livia@us.es (L.C.); ella.gorton@student.manchester.ac.uk (E.G.); patpergar2@alumn.us.es (P.P.-G.); 2 Biomedical Research and Innovation Institute of Cadiz (INIBICA), 11003 Cadiz, Spain; ricardo.pardillo@uca.es (R.P.-D.); ricardo.gomez@gm.uca.es (R.G.-O.); carmen.castro@gm.uca.es (C.C.); 3 Area of Physiology, School of Medicine, University of Cádiz, 11003 Cadiz, Spain

**Keywords:** oxidative stress, motor cortex, glutathione levels in the brain, postnatal development, membrane excitability, amyotrophic lateral sclerosis

## Abstract

Oxidative stress is one of the main proposed mechanisms involved in neuronal degeneration. To evaluate the consequences of oxidative stress on motor cortex pyramidal neurons during postnatal development, rats were classified into three groups: Newborn (P2–P7); infantile (P11–P15); and young adult (P20–P40). Oxidative stress was induced by 10 µM of cumene hydroperoxide (CH) application. In newborn rats, using the whole cell patch-clamp technique in brain slices, no significant modifications in membrane excitability were found. In infantile rats, the input resistance increased and rheobase decreased due to the blockage of GABAergic tonic conductance. Lipid peroxidation induced by CH resulted in a noticeable increase in protein-bound 4-hidroxynonenal in homogenates in only infantile and young adult rat slices. Interestingly, homogenates of newborn rat brain slices showed the highest capacity to respond to oxidative stress by dramatically increasing their glutathione and free thiol content. This increase correlated with a time-dependent increase in the glutathione reductase activity, suggesting a greater buffering capacity of newborn rats to resist oxidative stress. Furthermore, pre-treatment of the slices with glutathione monoethyl ester acted as a neuroprotector in pyramidal neurons of infantile rats. We conclude that during maturation, the vulnerability to oxidative stress in rat motor neurons increases with age.

## 1. Introduction

Oxidative stress is a pathological condition defined as the imbalance between harmful reactive oxygen species (ROS) and antioxidant defenses in an organism. ROS include free radicals, which are species that contain at least one unpaired electron on their outer shell, thus causing them to be highly reactive. ROS can be produced as a result of normal metabolic or inflammatory processes, as well as via external exposure to toxic substances [1]. The superoxide anion (O_2_^−^) is the most common ROS molecule found in the human body under physiological conditions, with the primary source of this anion being the mitochondria as a result of the leakage of electrons onto stable oxygen molecules during respiration. This anion can interact with hydrogen peroxide and nitric oxide to produce two other harmful intermediates: Peroxynitrate and the hydroxyl ion [2]. Cells have many endogenous antioxidant mechanisms to cope with these ROS species, with glutathione (GSH) being the main one, which is used by glutathione peroxidases to break down oxidants such as hydrogen peroxide [3,4]. At normal physiological levels, ROS have important functions as second messengers in many intracellular signaling cascades to maintain homeostasis. However, when ROS levels become notably elevated, the endogenous antioxidant defense fails, homeostasis cannot be maintained, and cells may undergo apoptosis. Cells are vulnerable to damage by ROS due to their lipid and protein membrane composition. These molecules can cause serious cell damage in several ways, including the opening of ion channels causing ionic imbalance, lipid peroxidation, and DNA and protein modifications, among others. Oxidative stress via ROS damage is implicated in many pathologies, including carcinogenesis, aging, diabetes, and atherosclerosis, as well as neurodegeneration [2,5,6,7]. The cortical accumulation of ROS species is associated with a gradual decline in brain function, as seen in many neurodegenerative disorders, such as amyotrophic lateral sclerosis (ALS) [8,9]. In relation to neurodegeneration, ROS have been shown to alter the electrical membrane properties of cells, including the cell membrane potential, ionic gradients, and cell excitability [10].

Cumene hydroperoxide (CH) and hydrogen peroxide are commonly used inducers of cellular oxidative stress in in vitro experiments for studying the mechanisms underlying oxidative damage [10,11]. CH can produce many different types of radicals, including alkoxyl radicals by the reduction of transition metals or lipid and lipid peroxyl radicals after a reaction with fatty acid side chains and oxygen. Radical-induced lipid peroxidation of membranes occurs as a result of CH application to biological tissues, producing oxidative stress [12]. Previous reports have used CH at a concentration of 10 µM to induce oxidative stress on the pyramidal neurons of the rat motor cortex to try to understand the role of oxidative stress in neurodegeneration [13]. The effects of oxidative stress in young adult rats (≥20 days) were investigated in a previous study by Pardillo-Díaz et al. [13]. Neurons were progressively depolarized without changes in the voltage threshold to elicit an action potential. At the beginning of the application of the oxidizing agent, the membrane resistance increased, and then subsequently decreased progressively after 15 min. This meant that the cells first suffered an increase in excitability, at the beginning of the application of the drug, which was revealed by a decrease in rheobase. Finally, after 30 min of CH application, 50% of the neurons were unable to discharge action potential repetitively, and the rest of the neurons, which maintained their firing properties, exhibited a diminished maximum frequency and gain of the frequency of discharge. In conclusion, oxidative stress had marked effects on the membrane properties, compromising both neuronal excitability and the ability to generate action potentials. 

The objective of this study was to ascertain whether oxidative stress affects the neurons of the primary motor cortex differently during postnatal development and if neurons’ ability to respond and defend against oxidative stress changes during maturation. 

## 2. Experimental Procedures

This work investigates the effects of CH on layer V pyramidal motor cortex neurons from newborn and infantile rats and compares them with the effects demonstrated in our previous study on young adult rats using the same animal handling and laboratory practices [13]. All procedures were conducted in strict accordance with the recommendations of the Guide for the Care and Use of Laboratory Animals of the European Community Directive 2003/65 and the Spanish Royal Decree 120/2005. The research protocol was approved by the Animal Ethics Committee of the Junta de Andalucía (03/05/2018/067). 

### 2.1. Animals and Preparation of the Brain Slices

Three groups of Wistar rats of both sexes, consisting of newborn rats (2–7 days old; P2–7), infantile rats (11–15 days old; P11–15), and young adult rats (20–40 days; P20–40), were studied separately. The newborn rats (*n* = 14) were sacrificed by direct decapitation, while the juvenile (*n* = 38) and young adult (*n* = 6) rats (>P7) were strongly anaesthetized (sodium pentobarbital, 50 mg kg^−1^) and perfused with ice-cold artificial cerebrospinal fluid (ACSF) with low calcium to slow down metabolism and achieve a higher rate of neuronal survival in the process of extracting and cutting the brain into slices, as previously described in detail [14,15,16]. The brain was rapidly extracted and placed in a Petri dish that contained ice-cold ACSF with low calcium. Using a scalpel, the cerebellum was removed, leaving the most rostral part of the brain, which contained the area of interest—the motor cortex. The tissue was cut transversely into 300–400 μm slices using a vibratome machine (NVLSM1, WPI). The slices were maintained in an ACSF-filled chamber at 33 degrees for 30 min, and then stored at ~21 °C in the same solution until use. The composition of the ACSF (mM) was as follows: 126 NaCl; 2 KCl; 1.25 Na_2_HPO_4_; 26 NaHCO_3_; 10 glucose; 2 MgCl_2_; and 2CaCl_2_. The concentrations (mM) for the low-calcium-ACSF were 4 MgCl_2_ and 0.1 CaCl_2_. Both ACSF and low-calcium-ACSF solutions were bubbled with 95% O_2_ and 5% CO_2_ (pH 7.4).

### 2.2. Whole-Cell Patch Clamp Recordings and Analysis

The brain slices were placed in the recording chamber, superfused with oxygenated ACSF via a pump (P-70, Harvard Apparatus) at 1 mL min^−1^, and warmed to 33 °C via a feedback-controlled heater (TC-324B, Warner Instruments Corporation). Visual guidance, including a Nikon Eclipse FN1 microscope with infrared-differential interference contrast (IR-DIC) optics, a 40× water immersion objective, and a WAT-902H2 Ultimate Camera, was used to patch clamp the motor neurons. Layer V pyramidal neurons were identified based on their location within the motor cortex and their unique morphology, consisting of a large projecting apical dendrite from the base of the pyramidal cell body [17]. Patch pipettes were obtained from a borosilicate glass capillary (inner diameter 0.6, outer diameter 1 mm; Narishige) using a puller (PC10, Narishige). Patch electrodes had a 3–5 MΩ resistance. For both current- and voltage-clamp experiments, the solution within the pipette was a K^+^-gluconate, where Na^+^ and K^+^ conductances remain functional so that neuronal firing can be assessed [18]. The composition of the K^+^-gluconate was as follows: 120 K-gluconate; 10 KCl; 10 phosphocreatine disodium salt; 2 MgATP; 0.3 NaGTP; 0.1 EGTA; and 10 HEPES. The pH was adjusted to 7.3 using KOH. An osmolarity of 285 mosmol/kg was maintained via adjustment with sucrose.

The formation of the whole-cell patch clamp was achieved using a micromanipulator (MP-225, Sutter) and an amplifier (Multiclamp 700B, Axon Instruments, Molecular Devices, Sunnyvale, CA, USA). Recordings were low-pass Bessel-filtered at 2–10 kHz; the data were digitized at 2–20 KHz with a Digidata 1550 analogue-digital converter and developed on a computer screen using the pCLAMP 10 software. Data were analysed using the Clampfit 10.4 software (Molecular Devices). In current-clamp mode, the bridge was periodically balanced using the auto-adjust feature. Throughout voltage-clamp recordings, the whole-cell capacitance was measured, and series resistances were compensated by 70%. Recordings were discontinued if the series resistance exceeded 20 MΩ.

### 2.3. Drugs and General Protocol

Each brain slice was first superfused with ACSF to acquire voltage/current-clamp recordings in a specific neuron in control conditions. Following this, to study the repercussion of the different drugs, the slice was superfused with ACSF containing the drugs and new recordings of the same neuron were obtained. Once we added CH to a slice, this oxidizing agent started irreversibly oxidizing all cells in the slice, so only one cortical neuron per slice was recorded. To obtain data on the effects of CH (Sigma-Aldrich, St Louis, MO, USA) on pyramidal neurons, cells were superfused with ACSF containing 10 µM for 30 min and readings were taken at 5, 15, and 30 min after CH application.

Drugs, except for CH, were purchased from Tocris Bioscience (Bristol, UK). All drugs were prepared in advance of the experiments in stock solutions dissolved in ACSF (1-10 mM) and stored at −20 °C. The drugs were mixed on the day of the experiment in ACSF with the following concentrations: 2-(3-carboxypropil)-3-amino-6-metoxyphenil-pyridazinium bromide (SR95531 o Gabazine, 20 µM); d-amino-phosphonovalerato (AP5, 20 µM); 6-cyano-7-nitroquinoxaline-2,3-dione (CNQX, 50 µM); 4,5,6,7-Tetrahydroisoxazolo [5,4-c]pyridin-3-ol hydrochloride (THIP, 1-2 µM); and glutathione monoethyl ester (4 mM).

### 2.4. Current- and Voltage-Clamp Recordings

To study the effects of CH on the intrinsic membrane properties, current-clamp experiments were performed, and the following parameters were measured: The resting membrane potential; input resistance; rheobase; voltage threshold for eliciting an action potential; depolarization voltage; action potential amplitude and duration; maximum frequency of discharge; frequency gain; and cancellation current. The methods employed for recording and analysing these membrane properties are fully detailed in previous works by our laboratory [19,20,21]. The resting membrane potential was the difference between the intracellular and extracellular potentials after moving the electrode away. To calculate the input resistance, positive and negative current pulses were injected into the cell (500 ms, 1 Hz; 10 pA each step), with the slope of the current–voltage relationship being the value of the resistance. Rheobase was considered the minimum current intensity (100 ms, 1 Hz; with 5 pA each step) able to produce an action potential in 50% of cases. The amplitude of the single action potential was measured as the difference between the voltage at a resting level and the voltage at the spike peak, while the duration was determined as the width of the spike at its half amplitude. The voltage increment in membrane potential required to achieve the voltage threshold was the depolarization voltage. To determine the spike threshold, the action potential recording was differentiated, with the spike onset taken as the value of the membrane potential at which the first derivative exceeded 10 V s^−1^. Repetitive discharge was evoked by depolarizing current steps (1 s, 0.5 Hz) with 10–50 pA increments. The firing frequency was taken as the number of spikes during the repetitive discharge. The slope of the relationship between the firing frequency and the current injected was the frequency gain. Additionally, the maximum firing frequency (highest frequency achieved by the neurons, regardless of the current intensity) and the cancellation current (minimum current required for the repetitive firing to cancel) were measured as described by Pardillo-Díaz et al. [13].

To demonstrate the presence of an inhibitory tonic current in pyramidal neurons of the primary motor cortex from infantile rats and investigate whether this tonic current was affected by CH, neurons were recorded at −70 mV. An intracellular solution based on CsCl was used to obtain ECl^−^ ~0 mV. The patch pipettes contained (in mM) 140 CsCl, 2 MgCl_2_, 0.05 EGTA, and 10 Hepes, adjusted to pH 7.3 with CsOH [22]. The amplitude of the tonic current mediated by GABA_A_ receptors was calculated as the difference between the level of control current and the level of current after the administration of a GABA_A_ receptor antagonist (Gabazine) and an agonist of extracellular GABA_A_ receptors (THIP). The change in the holding current amplitude after drug application was measured as the difference from the baseline level [22].

Finally, to investigate whether glutathione prevents the physiological changes induced by CH at 10 µM, slices of infantile rats were initially incubated for at least 1 h in normal ASCF with a 4 mM concentration of the membrane-permeable GSH derivative glutathione monoethyl ester (GSH-MEE; CAS 118421-50-4) [23,24]. Following this, pyramidal neurons (*n* = 12) were superfused with the same solution to measure electrophysiological parameters in current-clamp mode for the initial condition (time = 0). After this, each slice was superfused with ASCF, GSH-MEE, and 10 µM CH for 30 min, and voltage responses were recorded during this period.

### 2.5. Measurement of Lipid Peroxidation

Lipid peroxidation induced by CH was indirectly measured as the concentration of protein-bound 4-hydroxynonenal (4-HNE) in brain slices obtained from rats that were perfused with ice-cold ACSF, using identical procedures for all kinds of measurements (biochemical and functional parameters). Slices were incubated for 0, 5, 15, and 30 min with CH. After incubation, slices were weighed and 10–30 mg of tissue was homogenized in 7.5 mM phosphate buffer (2:1; *v:w*) containing protease inhibitors (Sigma Aldrich). Samples were then centrifuged at 14,000× *g* for 10 minutes and supernatant was used to detect protein-bound 4-HNE using the Lipid Peroxidation Assay Kit from Abcam, following the manufacturer’s instruction. A standard curve was constructed with BSA-bound 4-HNE. Results are given as ng of protein-bound 4-HNE/µg of protein.

### 2.6. Measurement of GSH and other Non-Protein Free Thiols in Brain Slices

To determine the cellular resistance to oxidative stress, we quantified the concentration of non-protein free thiol groups present in the sample. Brain slices including the motor cortex were extracted from rats of the three study groups (newborn, infantile, and young adult) and bathed in 10 µM CH for 5, 15, and 30 min. The free thiol content was measured as previously described by Castro et al. [24]. To measure the free thiol group content, slices were mechanically homogenized in 30 mM phosphate buffer (2:1; *v:w*). Homogenized samples were centrifuged at 16,000× *g* for 10 min to obtain the crude extracts. These supernatants were used immediately to quantify the concentration of protein-free thiols (with most of them being reduced GSH). Ellman’s reagent (dithio-bis-nitrobenzoic acid; DTNB) was used to measure the total protein-free thiol content of the samples. In order to eliminate protein thiols, proteins in crude extracts were precipitated using 70% ethanol (*v/v* final) and eliminated after a centrifugation step at 16,000× *g*, for 10 min. Measurements were performed on 6 µL of the supernatant in 94 µL of 100 mM potassium phosphate buffer (pH 7.5) containing DTNB at a final concentration of 1.5 mM. Thio-nitrobenzoate was assayed 5 min later by measuring absorbance at 412 nm. The absorbance of a standard curve using increasing concentrations (2.5–100 μM) of D,L-Homocysteine (r^2^ = 0.99) was used to extrapolate the absorbance values of the samples. Homocysteine was freshly prepared from homocysteine thiolactone, as previously described [25,26]. Standards were brought to 70% (*v/v*) ethanol to mimic the samples. The free thiol content was quantified as nmol of SH/mg of protein. The total protein concentrations in the homogenates were analysed using the Bradford Assay.

### 2.7. Measurement of the Glutathione Reductase (GR) Activity

The GR activity in brain slices was determined using the Glutathione Reductase Assay Kit from Abcam (ab83461; Abcam Plc., Cambridge, UK), according to the manufacturer’s instructions. The concentration of GSH formed from GSSG was analysed by its capacity to react with DTNB to produce TNB. Brain slices were mechanically homogenized and crude extracts were obtained by centrifugation at 16,000× *g*. Supernatant samples were incubated with DTNB and NADPH in the assay buffer. The change in absorbance at 405 nm was measured for 10 min and glutathione reductase activity was determined as the amount of TNB formed/min/mg of protein. The protein content was analysed by using the Bradford Assay.

## 3. Statistical Analysis

The results were expressed as the mean ± standard error, and *n* represents the number of cells used. To carry out the statistical calculations, GraphPad Prism was used. A normality test was carried out first to check the data distribution (Shapiro–Wilk test). An analysis of variance (ANOVA) with repeated measures was employed to compare the means of the time variable (0, 5, 15, and 30 min) for each electrophysiological parameter. This analysis was selected because the parameters were the same in number and from the same cells in each group. If the ANOVA test showed differences, the Bonferroni test was used to perform comparisons of groups. One-way ANOVA was also used to analyze the mean values of 4-HNE, free thiol content, and GR activity at 0, 5, 15, and 30 min for the three age groups (newborn, infantile, and young adult rats). If there were significant differences, the Bonferroni test was used again to perform a comparison of groups. Significant differences between currents after the application of gabazine and the application of CH or differences between currents after the application of gabazine and the application of gabazine plus CH were determined by using the Student’s *t* test for paired samples. Two groups of data were considered statistically significant if *p* ≤ 0.05. The asterisk (*) in figures and tables represents a significant difference from the control condition, while the cross (+) signifies a significant difference between two consecutive groups. The correlation between variables was measured by Pearson’s correlation coefficient (r).

## 4. Results

Layer V pyramidal primary motor cortex cells used for the recordings were selected according to their characteristic soma (pyramidal) and dendrite morphology (apical dendrite dorsally oriented). A preliminary experiment was carried out to ensure that neurons could maintain their intrinsic electrophysiological properties during a minimum of 30 min of recording to be certain that any changes observed in the properties were due to the effect of CH, rather than the effects of time. At this preliminary stage, cells (*n* = 6 of each group, newborn and infantile rats) were recorded for 30 min and no significant changes in their membrane properties were observed, as previously described in young adult rats [17].

### 4.1. CH Does Not Affect the Intrinsic Membrane Properties of Newborn Rats

Before administering the CH, the membrane potential of the sample group of pyramidal neurons (*n* = 15) was checked. Cells exhibited a stable resting membrane potential of −64.1 ± 1.7 mV and did not fire action potentials spontaneously. After 30 min of CH exposure, the membrane potential was −63.5 ± 2.4 mV, indicating that the membrane potential stayed the same and CH had a minimal effect on these newborn neurons.

**Figure 1:**
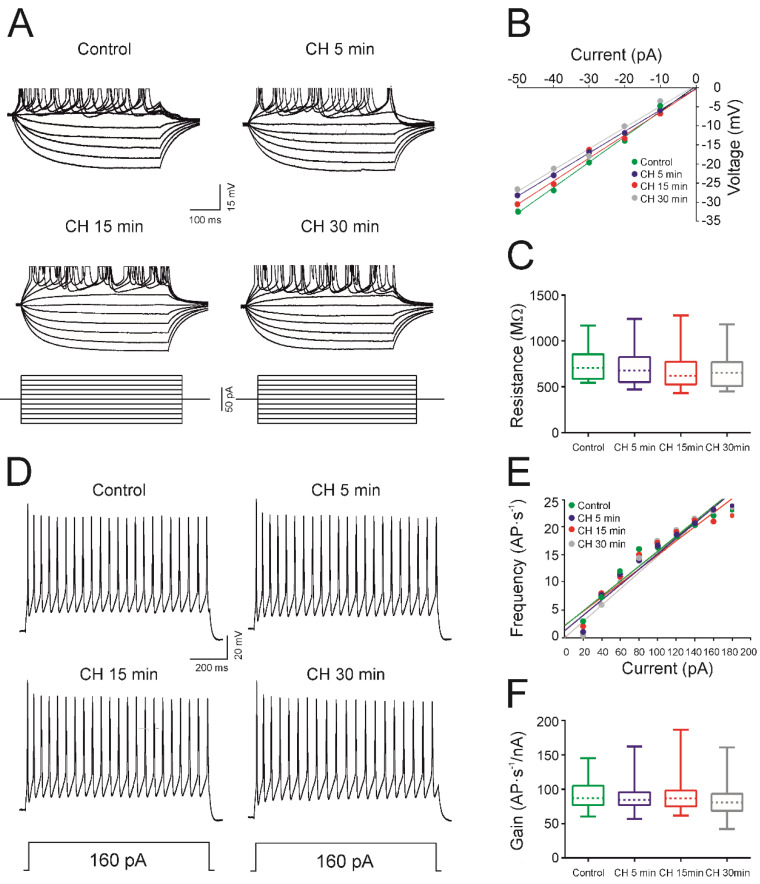
Effects of cumene hydroperoxide (CH) on the input resistance and firing properties in newborn rats. (**A**) Membrane voltage responses to depolarizing and hyperpolarizing current pulses in the same cell for control conditions and 5, 15, and 30 min after CH administration (10 μM). (**B**) Relationship between values of current and voltage represented in (**A**). (**C**) Effect of the CH on the membrane input resistance represented as a box and whisker plot showing the medians (dashed lines), interquartile ranges (boxes), and minimum/maximum values (whiskers) for respective datasets (*n* = 15). (**D**) Membrane potential responses to long-lasting depolarizing current pulses in the control situation and 5, 15, and 30 min after CH exposition. (**E**). Relationship between the current intensity and frequency of action potentials (AP) represented in (**D**). (**F**) Effect of the CH on the gain represented as a box-and-whisker plot showing the medians (dashed lines), interquartile ranges (boxes), and minimum/maximum values (whiskers) for respective datasets (*n* = 15). All recorded neurons were able to discharge repetitively in response to a depolarizing pulse of 1 s after CH application and no changes were observed in any of the electrophysiological parameters.

To observe the effects of CH on the neuron input resistance, depolarizing and hyperpolarizing currents were applied (Figure 1A) to the cells and the voltage–current (V–I) relationship was represented to calculate the resistance (Figure 1B). Figure 1A shows the voltage responses of a representative cell from the sample in the control condition and after 5, 15, and 30 min of CH exposure. There were no obvious observable changes in resistance when compared to the control condition. No significant differences among experimental groups were found (Figure 1C, Table 1).

To observe the effect of CH on the rheobase and depolarization voltage, current pulses were applied to the cell with increments of 5 pA, until the cell fired an action potential. The mean values for this experimental group showed no significant change in rheobase (Table 1). For the whole population (*n* = 15), the rheobase current was 34.7 ± 4.9 pA in the control and 42.7 ± 6.7 pA in the 30-min recordings. Table 1 also shows the mean values for the depolarization voltage and voltage threshold; no statistical differences were found. Additionally, in this study, no differences were found in the action potential amplitude or duration after exposure to CH.

Figure 1D shows the membrane response of a typical neuron from the sample in response to a long-lasting depolarizing current pulse (1 s) with a 160 pA intensity. As illustrated in this figure, no effects on the frequency of discharge were observed (control, CH 5 min, and CH 15 min, 22 AP·s^−1^; CH 30 min, 21 AP·s^−1^) and the repetitive discharge was maintained during the time of CH exposure, as in all recorded cells (*n* = 15). From the closeness and parallelism of the linear fits in Figure 1E, it can be inferred that no changes in frequency gain were obtained for the cell represented in 1D and for the whole population (Figure 1F and Table 1).

The maximum firing frequency and the cancellation current were also measured, and no changes were observed in response to CH administration (Table 1).

### 4.2. CH Does Affect the Intrinsic Membrane Properties of Infantile Rats

The cells of infantile rats (*n* = 15) showed a stable resting membrane potential of −64.5 ± 1.4 mV in the control condition and did not fire action potentials spontaneously. After 30 min of CH exposure, the membrane potential changed to −57.4 ± 1.5 mV, indicating that CH exposure produced a depolarization of the membrane potential, reaching statistical significance after 15 min of CH application (Table 2).

**Figure 2:**
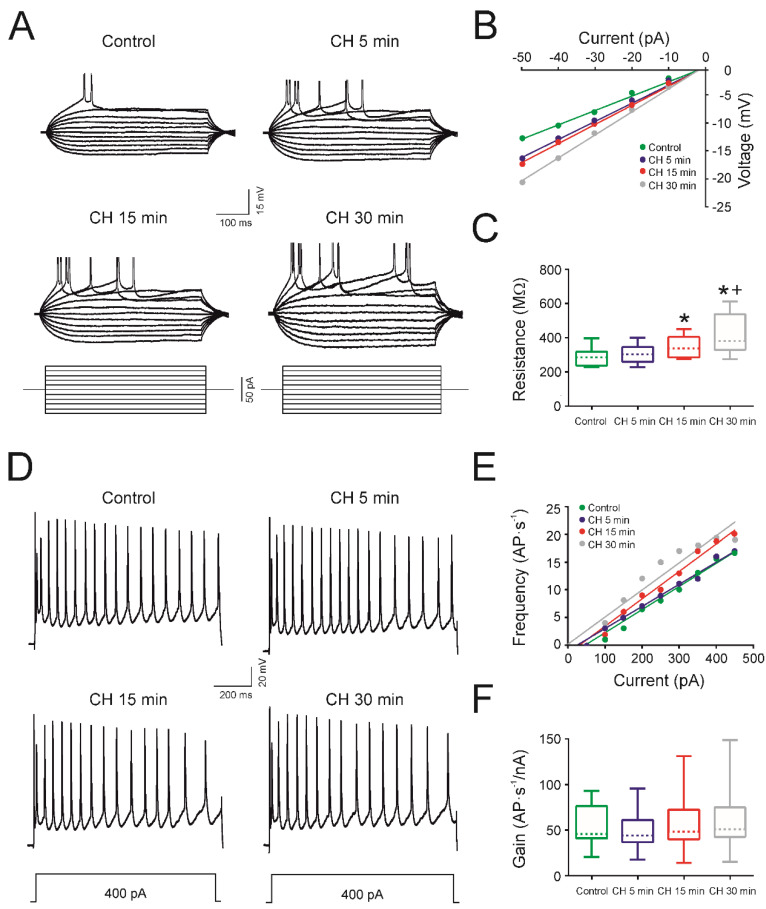
Effects of cumene hydroperoxide (CH) on the input resistance and firing properties in infantile rats. (**A**) Membrane voltage responses to depolarizing and hyperpolarizing current pulses in the same cell for control conditions and 5, 15, and 30 min after CH administration (10 μM). (**B**) Relationship between values of current and voltage represented in (**A**). (**C**) Effect of the CH on the membrane input resistance represented as a box and whisker plot showing the medians (dashed lines), interquartile ranges (boxes), and minimum/maximum values (whiskers) for respective datasets (*n* = 15). The box plot shows an increase in input resistance 15 min after CH administration. (**D**) Membrane potential responses to long-lasting depolarizing current pulses in the control situation and 5, 15, and 30 min after CH exposition. (**E**) Relationship between the current intensity and frequency of action potentials (AP) represented in (**D**). (**F**) Effect of the CH on the gain represented as a box-and-whisker plot showing the medians (dashed lines), interquartile ranges (boxes), and minimum/maximum values (whiskers) for respective datasets (*n* = 15). No changes in gain were detected after CH application. In (**C**), an asterisk indicates a significant difference from the control condition, and a cross indicates a difference between adjacent columns. The significance level was established as *p* ≤ 0.05.

The input resistance also changed after CH application (Table 2). Figure 2A shows the voltage responses of a representative motor cortex pyramidal cell in the control condition and after 5, 15, and 30 min of CH exposure. As depicted, the voltage response (and input resistance calculated from the V–I plot; Figure 2B) increased with time following CH exposure. The mean values of the input resistance for all neurons were 285.4 ± 12.7 MΩ in the control condition, and then increased to 302.2 ± 13.3 MΩ after 5 min, 348.0 ± 15.5 MΩ after 15 min, and 412.5 ± 29.8 MΩ after 30 min of CH exposure. Therefore, the input resistance increased, with CH exposure reaching statistical significance at 15 and 30 min compared to the control and being significantly different between 15 and 30 min of exposure (Figure 2C; Table 1). The mean values for the infantile rats also displayed a decline in rheobase (Table 2). The decrease in rheobase between the control and 5 min was not statistically significant (110.0 ± 14.7 vs. 93.0± 11.2 pA); however, between the control and 15 and 30 min, the decrease reached statistical significance. Additionally, a significant difference was found between 15 and 30 min of drug exposure (87.0 ± 11.0 vs. 72.0 ± 9.6 pA; Table 2). Table 2 also demonstrates that the mean values of depolarization and the threshold voltage exhibited no statistically significant changes.

In about 80% of the cells in the sample (12/15), the repetitive firing properties were maintained after drug application. In the group of cells that maintained their firing properties, CH produced no effects on the action potential amplitude and duration (Table 2). Figure 2D shows one of these neurons from the sample, presenting its firing properties in response to a depolarizing current pulse of 400 pA. The cell maintained its repetitive firing properties throughout the 5, 15, and 30 min of drug exposure, firing action potentials for the entirety of the 1 second pulse. In the control condition, the cell fired 19 action potentials, whilst 19 action potentials were recorded after 5 min of CH exposure, 16 action potentials after 15 min, and 16 after 30 min. The frequency gain was calculated from the F-I representation (Figure 2E) and no changes were found for the cell represented in 2D or for the whole population (Figure 2F and Table 2). The mean values for all experimental groups followed the same pattern (Table 2); no significant changes in gain were found (Figure 2F). However, a significant decrease was observed in the maximum frequency after 30 min of CH application and in the cancellation current after 15 min of CH application (Table 2).

### 4.3. CH Abolishes a Tonic GABAergic Current Mediated by GABA_A_ Receptors

In a previous report by our laboratory, we have shown that blocking the inhibitory synaptic transmission with gabazine (GABA_A_ antagonist) produces an increase in the input resistance of pyramidal neurons from young adult rats [27]. Under this condition of synaptic blocking, changes in membrane resistance were not observed in these neurons after CH exposure. These results allowed us to propose that the increase in resistance induced by CH in pyramidal neurons could be due to the involvement of a tonic inhibitory current mediated by GABA receptors.

**Figure 3:**
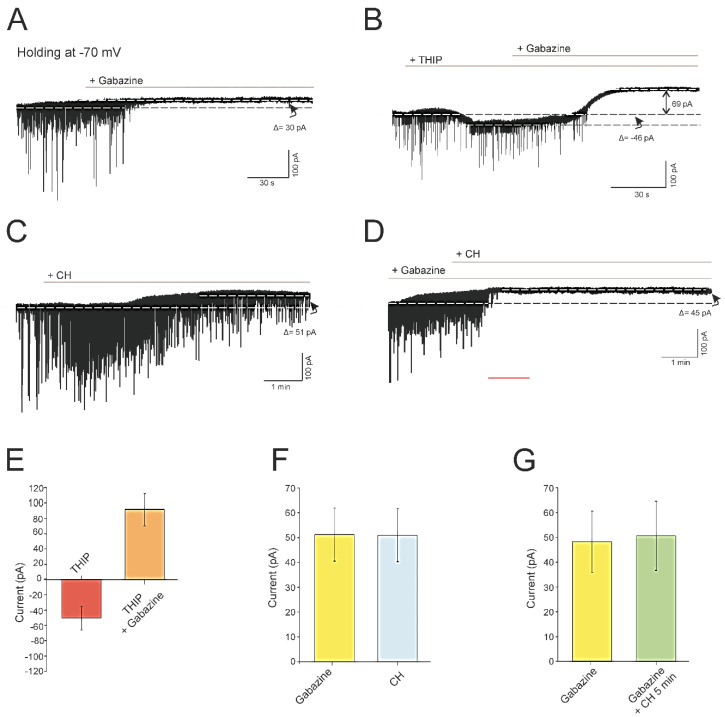
Effects of gabazine and cumene hydroperoxide (CH) on the holding current of pyramidal neurons from the motor cortex in infantile rats. (**A**) The application of gabazine 20 μM (*n* = 6) in the presence of glutamatergic antagonists blocked the spontaneous inhibitory postsynaptic currents (sIPSC) and revealed a tonic current mediated by the gabazine (GABA_A_) receptor (30 pA for the illustrated neuron). (**B**) Changes in the holding current after THIP application (an agonist of GABA_A_ extrasynaptic receptors) and after THIP plus gabazine in the same cell (*n* = 6). These experiments were carried out to check the presence of extrasynaptic receptors in this pool of neurons. (**C**) Effect of CH on the membrane current. Note that the application of CH in the presence of CNQX and AP5 produced an outward shift in the holding current when the holding potential was held at −70 mV (*n* = 6). (**D**) Effects on the membrane current during gabazine and CH consecutive application. Note that, in the same cell, no additional effects on the holding current were observed after CH administration. (**E**)–(**G**) Histograms showing the mean values (*n* = 6) of the changes in holding current after THIP and then after THIP plus gabazine in the same cells (**E**), after gabazine and after CH administration in different cells (**F**), and after gabazine and then after gabazine plus CH administration in the same cell (**G**). Note that no significant differences between both groups were found in (**F**,**G**). THIP, Tetrahydroisoxazolo [5,4-c] pyridin-3-ol hydrochloride; CNQX, 6-cyano-7-nitroquinoxaline-2,3-dione; and AP5, d-amino-phosphonovalerato.

To examine whether pyramidal neurons have a tonic GABA_A_ receptor-mediated current in a low ambient of GABA (without exogenous GABA), gabazine (20 μM) was delivered to the slice chamber in the presence of glutamatergic antagonists (CNQX, 50 μM; AP5, 20 µM) (*n* = 6, Figure 3A). Under these conditions, spontaneous inhibitory postsynaptic currents (sIPSC) were blocked, and an outward shift in the holding current was recorded when the holding potential was held at −70 mV. For the cell represented in Figure 3A, the shift in the holding current was 30 pA; the mean value for the whole population studied was 51.0 ± 10.7 (Figure 3F). This current was mediated by GABA_A_ receptors as it was blocked by the GABA_A_ receptor-antagonist gabazine. Therefore, we can conclude that even in the situations of low GABA concentrations, pyramidal neurons exhibit a tonic current that can be reversed by gabazine. To increase the current mediated by GABA_A_ receptors, THIP (GABA_A_ extrasynaptic receptor-agonist, 1 μM) was added to the bath and it produced an inward current carried by Cl^−^ (*E*CL = 0 mV) of −46 pA in the cell depicted in Figure 3B (average shift −48.2 ± 12.3 pA, *n* = 6, Figure 3E). The addition of gabazine to the recording chamber produced a strong outward shift in the holding current of a value of 115 pA in the same cell in Figure 3B (average for the whole population 91.2 ± 18.8 pA, Figure 3E). In the experiments in which CH was added to the bath, sIPSC decreased in frequency and amplitude in pyramidal neurons from infantile rats, as previously described for young adult rats [27], and an outward shift in the holding current was recorded, similar (not statistically different) to that produced by gabazine (Figure 3C,F). As shown in Figure 3C, the amplitude of the outward current was 51 pA (average 50.6 ± 10.7; *n* = 6, Figure 3F). Finally, we conducted another group of experiments (*n* = 6), in which we found that adding CH to the bath after gabazine did not produce any additional effect on the outward current (Figure 3D,G). All these results seem to indicate that CH reduces the inhibitory inputs received by pyramidal neurons of infantile rats, abolishing a tonic GABAergic inhibitory current mediated by GABA_A_ receptors. Therefore, reduced GABAergic inhibition underlies the increase in the excitability (membrane resistance) observed in pyramidal neurons of infantile rats after CH exposure, as suggested for pyramidal neurons of young adult rats [27].

### 4.4. Exposure to CH Induced Lipid Peroxidation

**Figure 4:**
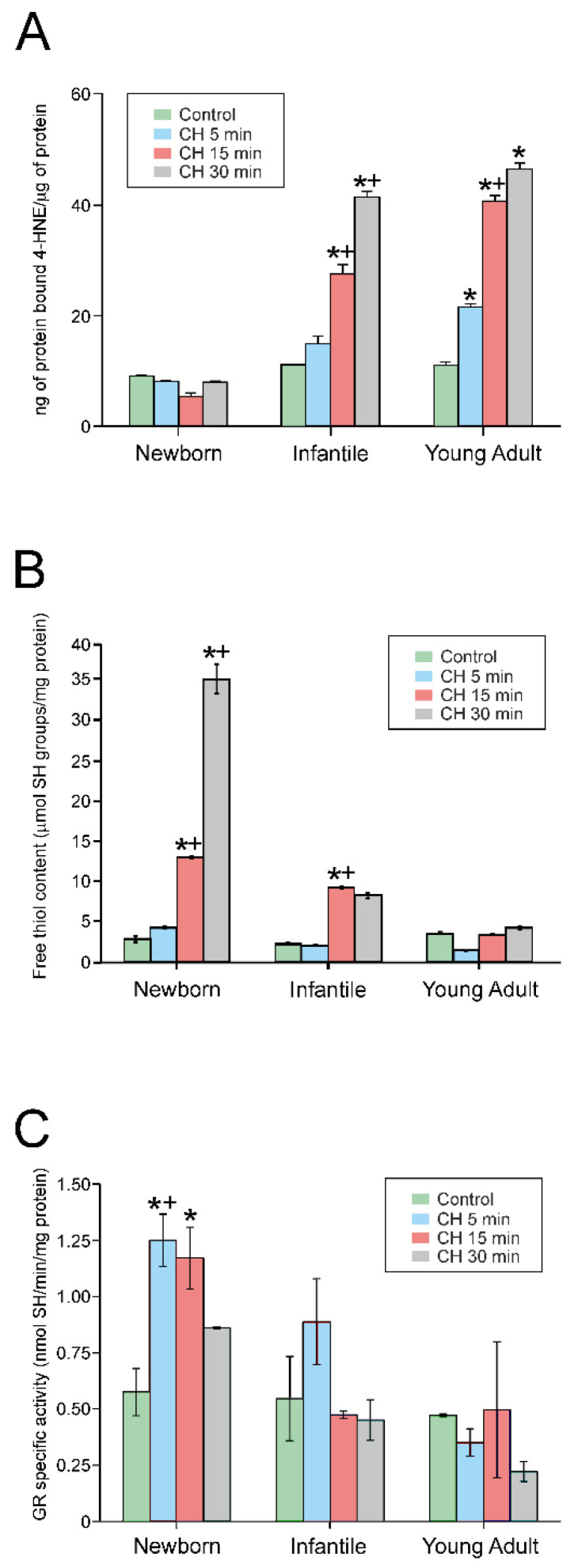
Antioxidant capacity of motor cortex neurons during development in response to cumene hydroperoxide (CH). (**A**) Lipid peroxidation expressed in ng of protein-bound 4-hydroxynonenal (4-HNE)/µg of protein. A bar chart representing the concentration of 4-NHE in the control condition and after 5, 15, and 30 min of 10 µM CH exposure in each experimental group (newborn, *n* = 6; infantile, *n* = 6; and young adult, *n* = 6) at 0 (green), 5 (blue), 15 (red), and 30 min (grey) after the exposure of CH. Note the increase in lipid peroxidation 15 min after the exposure to CH in infantile rats, and after 5 min in young adult rats. (**B**) Free thiol content in brain slices treated with CH. A bar chart representing the concentration of free thiols in the control condition and after 5, 15, and 30 min of CH exposure in each experimental group (newborn, infantile, and young adult). Note that neurons from newborn rats are more efficient at producing free thiols in response to CH than neurons from infantile and young adult rats. (**C**) Activation of glutathione reductase (GR) in response to the oxidative stress induced by CH. A bar chart representing the activity of glutathione reductase (nmol Sh/min/mg protein) in the control condition and after 5, 15, and 30 min of CH exposure in each experimental group. Note the increase in activity 5 min after the exposure to CH in newborn rats. In (**A**)–(**C**), * indicates a significant difference from the control condition, ^+^ indicates a difference between adjacent columns, and ^a^ represents a statistically significant difference between an age group and the precedent one (infantile vs. newborn and young adult vs. infantile) for the same period of time. The significance level was established as *p* ≤ 0.05. All data are presented as the mean ± standard error of the mean.

In order to understand the alterations in the response to CH in cells from different developmental stages, we measured lipid peroxidation (*n* = 6 in each experimental group). Figure 4A illustrates the variations in the concentration of 4-HNE protein adducts expressed as ng of protein-bound 4-NHE/µg of protein. The exposure of the brain slices to 10 µM CH for 5, 15, and 30 min increased the concentration of 4-HNE adducts when compared with the control situation (0 min) in all groups except for that of newborn rats. As shown in this figure, treatment with CH did not induce lipid peroxidation in this group. However, CH evoked lipid peroxidation, in a time-dependent manner, at 15 min in infantile rats and as soon as 5 min in young adult rats. Additionally, a significant difference was also found between 15 and 30 min of drug exposure in infantile rats, and between 5 and 15 min in young adult rats. Furthermore, at any time point, increments of lipid peroxidation were larger in infantile rats when compared to newborn rats, and larger in young adult rats compared with infantile rats.

### 4.5. CH Treatment Rapidly Increased the Free Thiol Content in Newborn Rats

We also measured the effect of CH on total non-protein free thiol concentration, mainly glutathione (GSH) (*n* = 6 in each experimental group). In control conditions (Figure 4B), the concentration of free thiols present in the slice homogenates was similar for all three experimental groups, and no statistical significance was observed between the control conditions. However, following 30 min of exposure to CH, the concentration of free thiols found in newborn rat homogenates was 3.5-fold greater than in infantile rat cells and 6-fold greater than in young adult rats. After 15 min of CH exposure, the concentration of free thiols produced by the newborn and infantile rat neurons was increased over the control and 5 min-exposure condition. After 30 min, the newborn rats significantly increased their free thiol concentration when compared to the other conditions and infantile and young adult rats (Figure 4B). This indicated that brain cells from newborn rats increased their free thiol content in response to CH, thus acquiring a greater resistance to oxidative stress.

### 4.6. CH Activates Glutathione Reductase in Newborn Rats after Treatment Initiation

We next tested whether the increase in free thiol levels was a consequence of the activation of glutathione reductase (GR) in response to the oxidative stress induced by CH, since this enzyme catalyzes the reduction of oxidized glutathione to produce free thiols. Therefore, we measured (*n* = 6 in each experimental group) the effect of incubation with CH on the GR activity of brain slices from newborn, infantile, and young adult rats (Figure 4C). The observed results indicate that in newborn rats, GR is activated 5 min after CH incubation and maintained after 15 min, but its activity returns to basal levels after 30 min of incubation. In infantile rats, although a tendency towards GR activation was observed at 5 min after CH incubation, it did not reach statistical significance. No effects on GR activation were observed in young adult rats. Furthermore, the GR activity induced by CH was significantly higher in newborn rats compared to infantile rats, and the GR activity was significantly higher in infantile rats at 5 and 30 min compared to young adult rats. These results may indicate that the cortex of newborn rats shows a higher redox buffering capacity than that of older rats.

### 4.7. Prevention of Membrane Excitability Changes Caused by Cumene Hydroperoxide by Glutathione Monoethyl Ester

**Figure 5:**
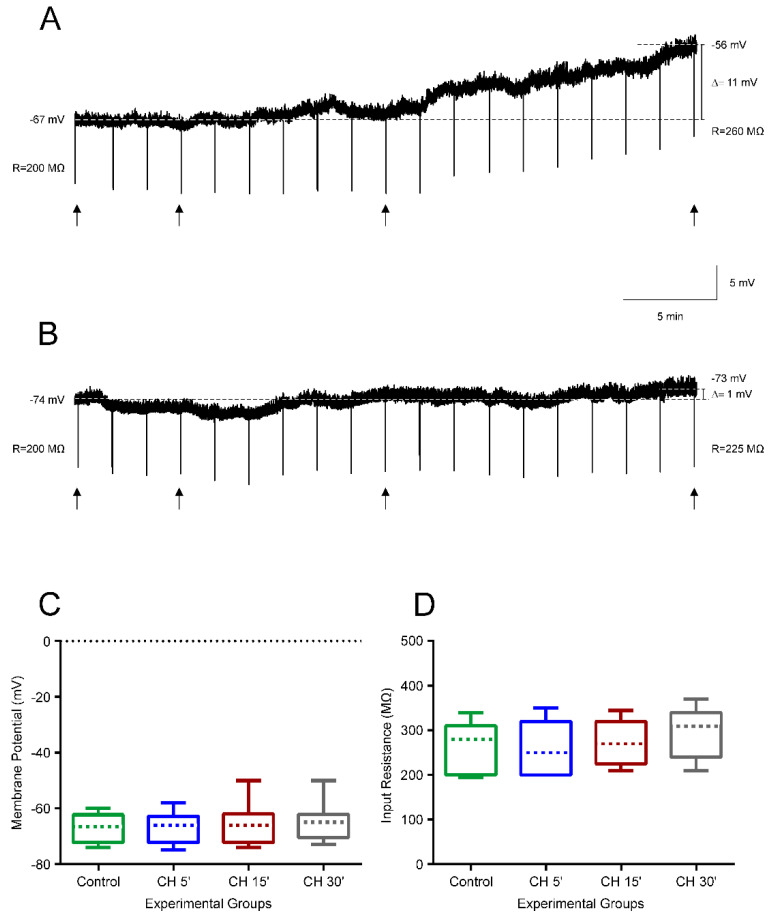
Prevention by glutathione monoethyl ester (GSH-MEE) of lipid peroxidation induced by cumene hydroperoxide (CH) on pyramidal neurons from the motor cortex in infantile rats. (**A**,**B**) Electrophysiological recording showing that the membrane potential depolarized and voltage response to negative current pulses of −100 pA and 500 ms increased throughout 30 min exposure to CH 10 µM (**A**) and remained stable (**B**) throughout 30 min exposure to CH 10 µM plus GSH-MEE (4 mM). Arrows indicate the voltage response at 5, 15, and 30 min. C,D. Box-and-whisker plot illustrating the medians (dashed lines), interquartile ranges (boxes), and minimum/maximum values (whiskers) of the membrane potential (**C**) and input resistance (**D**) in the control situation and 5, 15, and 30 min after CH exposure in slices preincubated with GSH-MEE (*n* = 12).

Our results indicate that brain cells from newborn rats increased their GSH content in response to CH, thus having a greater resistance to oxidative stress, which correlates with their ability to maintain their membrane electrical properties. To demonstrate that GSH prevents membrane alterations, we pretreated the infantile rat slices with 4mM GSH-MEE (a membrane-permeable GSH derivative), to observe whether it could act as a neuroprotector and prevent the electrical membrane alterations, induced by 10 µM CH, from occurring. Figure 5A illustrates the typical effects of 10 µM CH application on membrane properties for 30 min, in which the resting membrane potential depolarized and the input resistance increased. As can be seen in Figure 5B for a representative cell, no changes in membrane potential and input resistance were produced for the whole period when the slice was preincubated in 4 mM of GSH-MEE. No statistical differences were found in pyramidal neurons (*n* = 12) for any passive and active membrane properties measured after 30 min of CH exposure, when compared to the control value in the presence of GSH-MEE (Figure 5C,D).

## 5. Discussion

The main finding of this study, summarized in Table 3, was that the electrophysiological properties of newborn rat pyramidal neurons were largely unaffected by CH exposure throughout the 30 min recording, with no significant alterations. Additionally, some of the electrophysiological properties of the infantile rat pyramidal neurons, including the input resistance, rheobase, and maximum frequency, were significantly altered by CH exposure. In terms of the firing properties, 100% (15/15) of newborn rat neurons and 80% (12/15) of infantile rat neurons maintained their repetitive firing properties throughout the 30 min exposure. Our results indicate that newborn rats are completely resistant to the effects of oxidative stress, while infantile rats are partially resistant, with small alterations in some membrane properties. Greater alterations in membrane properties were observed in a previous study on young adult rats by Pardillo-Díaz et al. [13], suggesting that vulnerability to oxidative stress increases with age. Slices of newborn rats showed a greater ability to respond to oxidation by increasing their free thiol concentration, thus demonstrating a higher buffering capacity against oxidative stress. Finally, we have shown that pretreatment with GSH-MEE prevents the effects induced by CH on membrane properties.

### 5.1. CH as a Model to Study the Effects of Oxidative Stress

During respiration under normal physiological conditions, the mitochondrial electron flow produces the superoxide anion (O_2_^−^) from the reduced oxygen species that forms part of the ROS species. The superoxide ion can attack iron-sulfur-containing enzymes, producing another dangerous ROS molecule—the hydroxyl radical. These ROS species with an unpaired electron can attack cells and destroy cellular components, including lipids, DNA, and proteins, causing oxidative stress [28]. CH is an oxidizing agent that mimics the action of ROS species and is used in experimental studies to observe the effects of oxidative stress on cells. Hydrogen peroxide is another oxidizing agent used experimentally; however, it is required at lower concentrations of CH to produce oxidative stress [10,29]. In a toxicology study by Vimard et al. (2011), increased concentrations of CH were found to increase the death rate of the neurons, with this number becoming significant for concentrations over 10 µM. In a study by Pardillo-Díaz et al. [17], the effects of three concentrations of CH were investigated (1, 10, and 100 µM) and the effects of 100 µM did not vary significantly from those produced by 10 µM. However, using 10 µM, the cells could sustain their properties for longer, allowing a larger range of time to observe the changes in membrane properties that are induced by CH application. Additionally, Vimard et al. [30] found that, when exposed to CH for longer periods of time (60–180 min), the mortality rate of the cells was 70%. Therefore, in order to avoid cell death and prevent a complete loss of firing properties early, a concentration of 10 µM of CH was used and the cells were recorded for 30 min.

### 5.2. Influence of CH on the Passive and Active Membrane Properties

While, in newborn rats, no significant changes were found, some significant changes in the membrane properties of infantile rats were observed. A significant depolarization of the resting membrane potential was observed after 30 min of CH exposure, similar to that found in pyramidal neurons of young adult rats in response to CH [13]. Additionally, this finding is in agreement with the study on ventricular heart cells by Nakaya et al., in which CH produced a gradual depolarization in membrane potential [31]. This depolarization has been attributed to an inhibition in the activity of the inward rectifier K^+^ channel, which is predominantly involved in maintaining the resting membrane potential [32] or the depression of a leak background potassium current [33]. CH induced a slow developing inward current that caused membrane depolarization in pyramidal neurons of adult rats [13,27]. This effect has also been produced by hydrogen peroxide (1 mM; 30 min) in hypoglossal motoneurons [34]. These results contrast with those found in neurons in the paraventricular nucleus of the hypothalamus, where hydrogen peroxide induced membrane hyperpolarization via increased K+ conductance at the resting potential [35]. Pardillo-Díaz et al. [27] also showed that CH gradually inhibits synaptic GABA transmission in an acute slice preparation, which leaves neurons in their semi-intact network. Therefore, it could be proposed that when GABA transmission is gradually reduced by CH application, cellular hyperpolarization back to resting potential is prevented and therefore causes a gradual depolarization of the membrane potential. Furthermore, the resistance of the cells significantly increased at 15 and 30 min after CH application. This is in agreement with the results of a study by Nani et al. [34] on hypoglossal motoneurons, but in disagreement with a study on ventral horn neurons of the rat spinal cord [36], in response to hydrogen peroxide. Pardillo-Díaz et al. [27] also described a decrease in the frequency of spontaneous synaptic transmission, which suggests that oxidative stress induces changes in neurotransmitter release onto the pyramidal neurons, altering the efficiency of synaptic transmission. Previous reports described the lack of inhibitory inputs to be responsible for the increased hyperexcitability, identified by an increase in resistance, caused by hydrogen peroxide in the cells of the thalamocortical circuitry [27,37]. Therefore, it is possible that, in our study, CH may be progressively reducing GABAergic inhibition in infantile rats (present results) and in young adult rats [13], preventing the opening of Cl- membrane channels, and consequently producing an increased resistance and depolarization of the membrane potential.

Many studies on neurodegenerative disorders have observed a disruption of cortical interneurons, particularly GABAergic inhibitory interneurons. This is the case in ALS [38,39]. Sebe et al., in neocortical pyramidal neurons from newborn animals, demonstrated the presence of a tonic current activated by GABA that modifies neuronal excitability [40]. Another study, using a mouse model of ALS, showed that in layer V pyramidal neurons of the motor cortex, the input resistance increased as a consequence of a depression of the tonic GABA_A_ receptor-mediated current [41]. Therefore, reduced GABAergic inhibition might explain why pyramidal neurons from infantile rats (present study) and young adult rats [27] exhibited an increase in resistance under oxidative stress after CH exposition. We can conclude that CH induced an increase in membrane resistance influencing the tonic inhibitory current mediated by GABA_A_ receptors. Therefore, the overall effect of lipid peroxidation induced by CH was a depression in the input flow from the premotor interneurons that promote hyperexcitability of motor cortex pyramidal neurons of infantile and adult young rats.

In our study, CH exposure had a minimal effect on the repetitive firing properties of newborn and infantile neurons, which may be due to their increased resistance to the detrimental effects of CH on the membrane, indicating that lipid peroxidation is not as extensive. In total, 100% (15/15) and 80% (12/15) of the neurons from newborn and infantile rats maintained their firing properties throughout the 30 min of CH exposure, respectively. Comparing these values to those of young adult rat neurons studied by Pardillo-Díaz et al. [13], young adult rats were more vulnerable to the effects of CH, with only 57% (17/30) of cells maintaining their repetitive firing properties throughout the 30 min. Furthermore, the study by Pardillo-Díaz et al. [13] on young adult rat pyramidal neurons showed that CH exposure decreased the cancellation current and maximum frequency, as we also found in the present study in infantile rats. Therefore, due to oxidative stress, the cell becomes gradually less able to tolerate high current intensities, losing its repetitive firing properties earlier when compared to the control condition, resulting in a smaller maximum frequency. Our results suggest that Na^+^ channels may be largely unaffected, with most cells able to maintain their ionic gradients and uphold their repetitive firing properties in neurons of newborn and, and to a lesser extent, infantile rats. The cancellation of repetitive firing in 50% of the population of young adult rats [13] could be explained by a CH-induced reduced voltage-gated sodium current of the fast Na^+^ channels that are required for the activation of an action potential. As a result, the action potentials gradually get smaller in size until there is not enough Na^+^ flow to generate a spike, resulting in the loss of repetitive discharge. This phenomenon was previously described in myenteric [42] and hypoglossal neurons [34]. However, it is also likely that the reduction of other conductances could explain our results found in pyramidal neurons of young adult rats. It has been shown that oxidizing agents can affect the conductance of K^+^ voltage-gated channels, which could also contribute to alterations in the shape and reduction of the frequency of the action potentials [43,44,45,46]. Oxygen radicals produced by CH might directly attack ion channel proteins, or lipid peroxidation caused by CH might indirectly inhibit ion channel functions. Lipid peroxidation would alter the membrane lipid milieu surrounding the channel protein [31], modifying lipid–protein interactions [37].

### 5.3. Age-Dependent Vulnerability to Oxidative Stress

Neurons have various defense mechanisms against oxidative stress. Some of the compounds that are part of this defense are thiol-reducing agents, among which are N-acetylcysteine α or β-mercaptoethanol [29,47], or antioxidant substances, such as vitamin C and E and melatonin [12,17,48,49,50]. These antioxidants can prevent the alterations caused by oxidative stress, including the peroxidation of membrane lipids and oxidation of mitochondria and nuclear DNA [50,51,52,53]. Our results showed a time-dependent increase in 4-HNE induced by CH treatment in all groups except for that of the newborn rats. HNE is a product of lipid peroxidation, which modifies proteins reacting with an amino group or with a protein side chain, thus leading to protein damage [54]. GSH plays an important role in eliminating the HNE-induced effects of lipid peroxidation. Therefore, 4-HNE protein modifications may be reverted in the presence of adequate concentrations of free GSH, especially cysteine modifications [55]. Interestingly, we observed a 3.5-fold increase in the concentration of free thiols (mainly GSH) in newborn rats in comparison to the infantile rats and a 6-fold increase compared to young adult rats after 30 min of CH exposure. GSH is the most abundant non-protein free thiol found in cells and plays a significant role in various endogenous pathways to fight ROS species and resist conditions of oxidative stress. Our study shows that the older motoneurons were much less able to reduce glutathione in response to CH exposure, suggesting that the mechanisms involved in the antioxidant system may become damaged with age. Moreover, we also showed that GSH-MEE protects neurons from infantile rats from the CH-induced alteration of their electrical properties, thus corroborating the protective role of GSH production in newborn rats. To coincide with our study, Deepashree et al. observed an age-related decline in antioxidant defenses following ethanol-induced oxidative stress on *Drosophila* [56] and Guevara et al. detected a significant age-related reduction in antioxidant enzyme activities, including superoxide dismutase and catalase, in rat brains [57]. Previous reports suggest that an age-dependent increase in mitochondrial dysfunction may cause the decrease in resistance to oxidative stress [28,58]. In support of this, the age-related downregulation of many drosophila genes, involved in metabolism and the antioxidant system, have been observed. Transcript levels of many key genes involved in the mitochondrial electron transport chain, such as cytochrome C and ATP synthase, became reduced with age [59]. As well as a decrease in ATP production, an impairment in the electron transport chain also leads to a greater production of ROS which further damage mitochondrial DNA and proteins. The brain requires energy in the form of ATP from the mitochondria for normal functioning. Therefore, a link can be made between oxidative stress and ROS production, impaired mitochondria, and an increased tendency towards neurodegeneration patterns observed in diseases such as ALS [58,60]. In agreement with our results, defective antioxidant defenses, cytoplasmic nucleic acid oxidative damage, and altered redox status sensors have been found in neurons of murine Alzheimer’s disease models, from 3 months of age, indicating that oxidative stress is an early pathogenic factor. In our study, we showed that the redox buffering capacity starts to be compromised as early as 15 days of age in comparison with neonatal rats [61]. Furthermore, supporting our findings, physiological reducing agents such as GSH seem to participate in other neurodegenerative disorders. Hence, the GSH buffering system has been proposed to control oxidative stress in Parkinson’s disease (PD). Murine models of PD show a reduced GSH metabolism and a reduced GSH/GSSG ratio triggered by oxidative stress, which constitute critical factors in the oxidative and neuroinflammatory processes associated with this particular neurodegenerative disorder [62].

In the mammalian brain, CH is generally detoxified in astroglial cells by the GSH system, producing GSSG. The GSSG produced in vivo during the reaction catalyzed by GPx is reduced by glutathione reductase, which is an enzyme that uses NADPH as a co-substrate. Therefore, the detoxification of peroxides is directly linked to glutathione reductase activity [63,64]. In the current study, we found that only neurons from brains from newborn rats respond to CH, by increasing their redox buffering capacity.

We can conclude that cellular resistance to oxidative stress is age-dependent. Oxidative stress induced by CH has no effect on newborn rats due to the induction of a relatively more powerful antioxidant defense system. CH produces physiological alterations in membrane properties in infantile rats, which become more severe with age. During postnatal development, age-related reductions in enzymatic activity linked to metabolism and general cellular function occur, resulting in an increased production of ROS and limiting the internal ability of the cells to maintain their antioxidant defense system. This inability to produce antioxidants is not only a feature of the ageing brain, but also of neurodegenerative disorders such as ALS, in which oxidative stress greatly contributes to its pathogenesis. Therefore, we hypothesize that damage caused by oxidants such as CH mainly contributes to neuronal dysfunction in aged brains, and therefore, neurodegenerative diseases such as ALS are more likely to occur in the older population.

## Figures and Tables

**Table 1:** Effects of cumene hydroperoxide (CH) on the electrophysiological properties on the pyramidal motor cortex neurons of newborn rats.

Membrane Properties	Control	CH 5 min	CH 15 min	CH 30 min
Membrane potential (mV)	−64.1 ± 1.7	−63.9 ± 2.0	−64.4 ± 2.2	−63.5 ± 2.4
Input resistance (MΩ)	744.6 ± 46.1	710.5 ± 50.5	675.3 ± 55.1	665.8 ± 49.3
Rheobase (pA)	34.7 ± 4.9	36.0 ± 4.0	40.3 ± 4.9	42.7 ± 6.7
Voltage depolarization (mV)	21.7 ± 2.0	21.1 ± 1.6	23.5 ± 2.1	22.77 ± 2.2
Voltage threshold (mV)	−43.5 ± 2.8	−44.5 ± 2.6	−42.3 ± 2.3	−41.5 ± 2.4
Action potential amplitude (mV)	92.4 ± 1.0	91.1 ± 1.6	91.3 ± 1.7	89.7 ± 2.0
Action potential duration (ms)	2.98 ±0.16	3.03 ±0.18	2.95 ±0.18	3.01 ±0.22
Gain (AP·s^−1^·pA^−1^)	91.1 ± 6.4	90.0 ± 7.4	93.3 ± 9.1	85.6 ± 8.8
Maximum frequency (AP·s^−1^)	18.1 ± 1.4	18.6 ± 2.0	17.8 ± 2.0	16.7 ± 1.4
Cancellation current (pA)	174.2 ± 17.2	192.9 ± 14.7	186.2 ± 15.9	173.3 ± 17.3

A cross indicates a significant difference from the control condition; an asterisk indicates a difference between adjacent columns. The significance level was established as *p* ≤ 0.05. All data are presented as mean ± standard error of the mean.

**Table 2:** Effects of cumene hydroperoxide (CH) on the electrophysiological properties on the pyramidal motor cortex neurons of young rats.

Membrane Properties	Control	CH 5 min	CH 15 min	CH 30 min
Membrane potential (mV)	−64.5 ± 1.4	−63.5 ± 1.5	−58.3 ± 1.1 *+	−57.4 ± 1.5 *
Input resistance (MΩ)	285.4 ± 12.7	302.2 ± 13.3	348.0 ± 15.5 +	412.5 ± 29.8 *+
Rheobase (pA)	110.0 ± 14.7	93.0 ± 11.2	87.0 ± 11.0 +	72.0 ± 9.6 *+
Voltage depolarization (mV)	23.9 ± 1.5	23.1 ± 1.3	21.4 ± 1.7	20.9 ± 1.9
Voltage threshold (mV)	−42.0 ± 1.1	−41.2 ± 1.0	−38.7 ± 1.5	−37.9 ± 2.2
Action potential amplitud (mV)	104.1 ± 2.9	100.4 ± 3.1	98.5 ± 2.8	97.6 ± 2.3
Action potential duration (ms)	1.96 ± 0.11	2.15 ± 0.11	2.13 ± 0.11	2.04 ± 0.10
Gain (AP·s^−1^·pA^−1^)	53.8 ± 7.0	49.9 ± 7.1	57.6 ± 9.7	60.7 ± 11.1
Maximum frequency (AP·s^−1^)	20.6 ± 2.3	20.2 ± 2.4	18.7 ± 2.3	17.5 ± 2.0 *
Cancellation current (pA)	437.0 ± 64.6	457.0 ± 71.7	345.0 ± 48.9 *+	302.0 ± 42.6 *+

A cross (+) indicates a significant difference from the control condition; an asterisk (*) indicates a difference between adjacent columns. The significance level was established as *p* ≤ 0.05. All data are presented as mean ± standard error of the mean.

**Table 3:** Summary of the differential effects of CH on motor cortex pyramidal neuron membrane properties according to age group.

Membrane Properties	Alteration (Minimum Incubation Time Required)		
	Newborn	Infantile	Young Adult
Membrane potential	=	↑ (15 min)	↑ (5 min)
Input resistance	=	↑ (15 min)	↑ (5 min) ↓ (15 min)
Rheobase	=	↓ (15 min)	↓ (5 min) ↑ (15 min)
Voltage depolarization	=	=	↓ (5 min)
Voltage threshold	=	=	=
Action potential amplitude	=	=	↓ (5 min)
Action potential duration	=	=	↑ (5 min)
Gain	=	=	↓ (15 min)
Maximum frequency	=	↓ (30 min)	↓ (5 min)
Cancellation current	=	↓ (15 min)	↓ (15 min)

Table indicates the alterations found (↑: increase, ↓: decrease, and =: no alteration) and the minimum time of incubation with CH required to observe the alteration.
